# Epidemiology and effect of gastrointestinal nematodes on dairy goats in Argentina

**DOI:** 10.4102/ojvr.v84i1.1240

**Published:** 2017-02-28

**Authors:** Victor H. Suarez, Gabriela M. Martínez, Alberto E. Viñabal, José R. Alfaro

**Affiliations:** 1 Instituto Nacional de Tecnología Agropecuaria – AISA-IIACS, Centro de Investigaciones Agropecuarias, Argentina

## Abstract

The aim of this work was to study the epidemiology and harmful effects of gastrointestinal nematodes (GINs) on dairy goats maintained in an intensive system. Two groups of goats were studied: untreated group (UG) (subdivided into UGjun goats that kidded in June, and UGjul goats that kidded in July) and treated group (TG) (with no subgroups, treated with monepantel: 3.75 mg/kg, orally, monthly). Eggs per gram (epg) in faeces were counted, faecal culture was performed to differentiate nematode genera and milk production was measured. Differences between groups were compared using least squares means analysis of variance (milk production and milking period length) and Kruskal–Wallis test (faecal egg counts). Nematode infection was moderate, with *Haemonchus* and *Trichostrongylus* being the dominant genera; the faecal egg counts reached the level of 2000 only once throughout the study. Goats that kidded in June had higher egg count after parturition (UGjun = 1564 epg), with significant differences (*p* < 0.04) from those that still had not kidded (UGjul = 962 epg). Over the entire trial period, the mean total milk production of TG (399.5 L ± 34.0 L) was significantly higher (*p* < 0.05) than that of UG (281.6 L ± 37.5 L), representing an increase of 41.8% in total milk yield. The results of this study show a post-partum peak in egg count and a negative effect of GINs on milk yield, even with moderate infections.

## Introduction

Goat production in the Argentine provinces of Salta and Jujuy is mostly meant for milk production and commercialisation or cheese production for on-farm consumption. This activity is based largely on extensive goat exploitation and, to a lower degree, on dairy industries that have invested in capital and inputs, and are based on more intensive systems. These undertakings are generally based on dairy breeds such as Saanen and Anglo-Nubian or their crosses with criollo goats and have unresolved problems regarding primary production. Among these production concerns, health problems are a very important limiting factor.

One of the health issues is the infection by gastrointestinal nematodes (GINs). This is a very important factor limiting goat milk production in Argentina, affecting economic competitiveness and even causing high animal mortality (Aguirre et al. 2002; Suarez et al. 2013). These works, as well as others from northeastern Córdoba and Mendoza (Anziani et al. 2010; Dayenoff et al. 1996), recognised *Haemonchus contortus* as the most harmful species. However, at the start of autumn, the burdens of *Trichostrongylus colubriformis* severely affect health and milk yield in goats (Suarez et al. 2013). Despite this background information and other data on the development of anthelmintic resistance (Aguirre et al. 2000; Suarez et al. 2012), baseline epidemiological information is still lacking for elaborate control strategies in the diverse goat productive systems and environments in northwestern Argentina, as well as in the irrigated temperate valleys.

The effects of GINs on overall production and especially on milk production of dairy goats have not been deeply studied worldwide (Hoste & Chartier 1998; Rinaldi, Veneziano & Cringoli 2007). In Argentina, studies on small ruminants include some works about sheep in La Pampa province (Suarez, Cristel & Busetti 2009) and goats in San Luis province, which describe the effect of parasites on milk production of criollo dairy goats (Rossanigo, Frigerio & Silva Colomer 1999). In Salta province, Suarez et al. (2013) observed a 15% increase in milk production in response to an anthelmintic treatment with respect to an untreated group (UG). The aim of this work was to contribute to the knowledge on epidemiology and effects of GINs on goat milk production in the irrigated fields of temperate highland valleys in northwestern Argentina over a lactation period.

## Materials and methods

### Study site and flock management

The study was conducted at the Dairy Goat Unit of Salta Agricultural Experimental Station, located in Valle de Lerma, northwestern Argentina, at 1050 m a.s.l., from June 2013 to April 2014. Rainfall in this temperate valley is concentrated in summer, with a dry period from April to November. Mean annual rainfall is 800 mm and mean temperature is 17 °C (maximum: 36 °C; minimum: -6 °C), with relative humidity between 20% and 80%.

The study flock comprised mostly Saanen goats. Forage management before the start of the experiment was based on lucerne grazing. Then, 1 month before kidding, goats were housed and fed on a ration of sorghum silage, hay and maize grain, until mid-July, when they grazed on oat with vetch. By mid-August, they were housed again until the end of October; after that, they grazed again on oat pastures, and then on lucerne. In February they grazed on sorghum and by mid-March they grazed on lucerne, until the end of the study. Pastures were grazed by 10 goats per hectare from May to November and were irrigated during winter by a furrow system.

Milk control started 4 days after parturition, after the kids were weaned. The milking system was mechanical and once daily, and goat feeding was supplemented with 300 g of maize.

### Experimental design

Parasite infection and milk production were monitored in 36 milking goats naturally infected with GINs between June 05, 2013 and April 17, 2014. These goats were synchronised and mated during December and January. Two groups of goats were formed: UG (*n* = 21), subdivided in two subgroups: UGjun goats (kidding in June, *n* = 10) and UGjul goats (kidding in July, *n* = 11). These subgroups were studied until November 05; after that, they were considered a single UG. The other group was the treated group (TG) (*n* = 15) (treated with monepantel: 3.75 mg/kg, orally, monthly), with no subgroups, formed with the sole purpose of obtaining an optimal group without parasites to compare the nematode effect and not to propose it as a method of control.

The unequal number of TG goats was to prevent the alteration of the number of pasture nematode larvae and to prevent anthelmintic resistance. Those untreated animals that exceeded 3000 epg or showed clinical symptoms of nematode infection received monepantel (3.75 mg/kg) orally.

### Parasitological measurements

Individual faecal nematode egg counts were determined monthly using the modified McMaster technique (Roberts & O’Sullivan 1949). Faecal cultures were similarly performed monthly per goat group to assess the generic composition of nematode populations according to Suarez (1997). Two UG goats died during the trial and necropsy procedures and worm burden were estimated according to Suarez (1997).

### Evaluation of production

Milk yield per goat was measured weekly from the start of milking until the dry period and the end of lactation. Milk yield was estimated following Fleischmann method (ICAR 1992) using New Zealand goat milk meters (Waikato MKV Milk Meter).

### Statistical analysis

Least squares means analysis of variance was conducted using the mixed models procedure of InfoStat Statistical Software (Di Rienzo et al. 2008). The following fixed effects were included in the models for milk production and milking period length per goat as explanatory variables: treatment group (TG and UG), type of birth (triplets, twins or single) and the interactions of treatment group with type of birth. Goat parity number was used as co-variable. Differences were compared using the Tukey test. Faecal egg counts were tested with non-parametric Kruskal–Wallis test.

In the months of February, March and April, the proportion of goats of each group that remained in milk and those that were removed from milking because their daily milk production dropped to less than 200 mL was analysed by Chi-square analysis using Fisher’s test.

## Results

### Parasitological parameters

**FIGURE 1:**
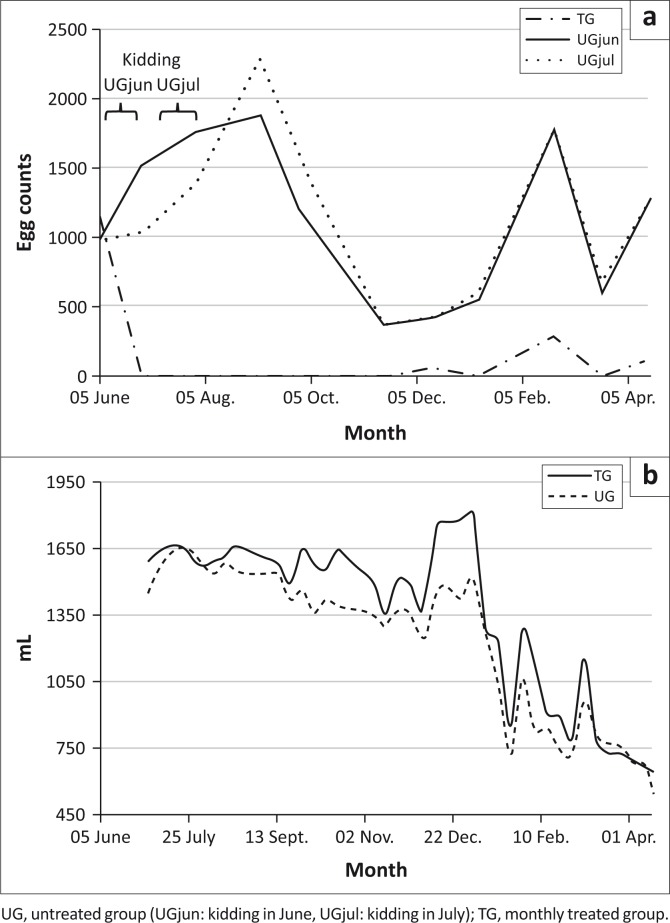
Mean faecal (a) egg counts and (b) mean monthly milk production per group during the experiment period.

Figure 1 shows worm faecal egg counts of groups throughout the trial. UGjun subgroup showed significantly higher (*p* < 0.04) egg counts after kidding than non-parturient goats (UGjul). Then, after July kidding, egg counts of the UGjul subgroup showed similar post-partum peak and values to those of UGjun. Towards November, after a period when goats were housed (Aug. 02 – Oct. 21) egg counts dropped to low levels, which persisted for 80 days. In January, after heavy rainfalls, egg counts of UG group increased significantly (*p* < 0.0001), reaching a peak in February. Those egg count levels sharply declined to 633 epg in March, when goats were fed on annual sorghum. Then mean egg counts of the UG group increased again when lucerne pasture was consumed by goats until the end of the experiment.

During the whole trial, the goat flock was in good body condition, except in September, December and February, when four, one and three does of UG group, respectively, had to be dewormed because they presented high egg counts and poor condition. After the first treatment, the TG group had negligible faecal egg counts throughout the trial, showing low peaks in December, February and April.

**FIGURE 2:**
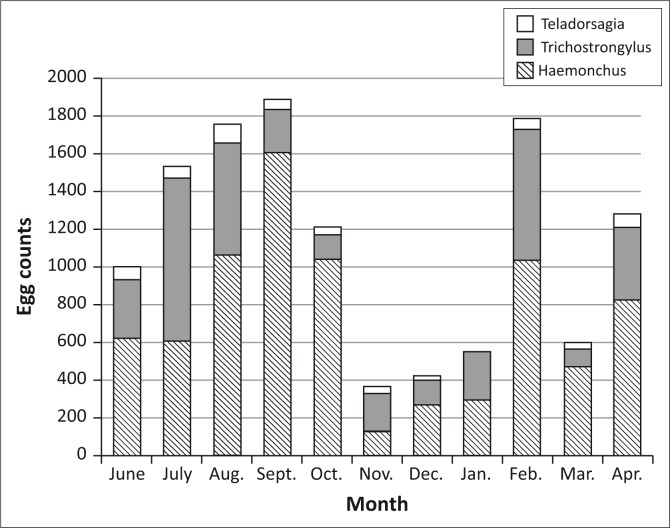
Nematode genera identified from culture of goat faces and proportion of each genus as detected from mean egg count of untreated group during the trial.

*Haemonchus* sp. and *Trichostrongylus* sp. were the dominant nematode genera recovered from faecal cultures. Figure 2 shows the periodic variation of proportion of prevalent nematode genera during the trial. *Haemonchus contortus, T. colubriformis, Teladorsagia circumcincta* and *Trichuris* sp. were recovered from the two necropsied goats.

### Milk production

Figure 1 shows the milk production of the goat groups during the study. Over the entire trial period, the mean total milk production (TMP) of TG (399.5 L ± 34.0 L) was significantly higher (*p* < 0.05) than that of UG (281.6 L ± 37.5 L), representing an increase of 41.8% in total milk yield. The mean daily milk production per goat of TG (1.51 L/day ± 0.10 L/day) was higher (*p* < 0.05) than that of UG (1.25 L/day ± 0.12 L/day). Throughout the trial, mean milk yield values showed differences between groups during the post-parturient period (*p* < 0.05) in August, at the beginning of December (*p* < 0.09) and early February (*p* < 0.02). Table 1 summarises milk production data.

**TABLE 1:** Mean (± SEM) and ranges of total milk production, mean daily milk production and machine-milking period length for treated and untreated groups.

Variables	Groups	Mean (±SEM)	Minimum	Maximum
TMP (L)	UG	281.60 ± 37.50^a^	180.10	535.20
	TG	399.50 ± 34.00^b^	215.00	560.00
MMD (L/goat/day)	UG	1.25 ± 0.12^a^	0.84	2.17
	TG	1.51 ± 0.10^b^	0.91	1.93
MML (days)	UG	223.30 ± 10.80^a^	226.00	292.00
	TG	262.30 ± 9.80^b^	138.00	290.00

Means with different letters differ significantly (*p* < 0.05).

MMD, mean daily milk production per goat; MML, machine-milking period length; TG, treated; TMP, total milk production; UG, untreated.

The machine-milking period length (MML) of the TG group (262.3 ± 9.8 days) was significantly (*p* < 0.041) longer than that of the UG group (223.3 ± 10.8 days). The other explanatory factor that was found to be significant in the linear mixed model of overall milk production parameters was goat type of birth for TMP. There was no significant interaction between treatments (TG and UG) and goat type of birth, nor was there any effect on goat parity number. The productive response to treatment was independent of the mentioned variables.

In March, the number of does of the UG group, whose daily milk production dropped to less than 200 mL and thus had to be withdrawn, was higher (Chi-square 4.66; *p* < 0.03) than that of the TG. Table 2 illustrates the number of does of each group that were removed from the trial in February, March and at the end of the milking period in April.

**TABLE 2:** Contingency tables of the number of goats that remained in milk and goats that were removed from the trial because their milk production declined below the threshold level.

Date	Group	Number of goats remaining	Number of goats removed	Chi-square
February 13	UG	15	6	3.33 (*p* < 0.07)
	TG	14	1	
March 13	UG	13	8	4.66 (*p* < 0.032)
	TG	14	1	
April 17	UG	2	19	4.70 (*p* < 0.03)
	TG	6	9	

UG, untreated; TG, monthly treated.

## Ethical considerations

The study has been approved by the Ethical Review Committee (CICUAL: Institutional Committee of Care and Use of Experimental Animals) of the University of La Plata, Argentina.

## Discussion

In agreement with previous reports for the region, *H. contortus* and *T. colubriformis* were the most prevalent nematode species, with *T. circumcincta* being less prevalent (Suarez et al. 2013). Significant differences in faecal egg counts between the UGjun lactating goat subgroup and the UGjul pregnant goats indicate a post-partum peak in egg count (Figure 1). In a previous assay, Suarez et al. (2013) observed that goats kidding in late winter (September) exhibited higher egg counts than goats that had kidded 3 months before, but under different management practices. Our results indicate that the post-partum peak in egg count was a result of goats being housed and supplied with the same forage type. The egg count recorded at the start of the assay would be owing to nematode infection that occurred before the beginning of the present study, since goats had been housed 30 days before parturition and had no possibility of ingesting infesting larvae. At this stage, a phenomenon known as periparturient rise of epg occurs in sheep and goats owing to relaxation of immunity triggered by a rise of prolactin at the start of lactation. This epg peak was well described for sheep in several parts of the world (Armour 1980; Connan 1976), as well as in Argentina (Suarez 2007; Suarez & Busetti 1995); however, this periparturient rise of egg count has been scarcely mentioned in goats (Mandonnet et al. 2005). In San Luis, Rossanigo and Frigerio (2000) observed a tendency of increasing epg in dairy goats during the spring–summer kidding periods (November–December) and at the end of autumn (May–July). Rahman and Collins (1992) noted that mean weekly egg counts of pregnant goats were significantly higher than those of non-pregnant goats and that mean egg counts and prolactin concentration of pregnant goats during the 6-week post-partum period was significantly higher than during the 6-week pre-partum period.

In general, GIN burdens were low to moderate (Skerman & Hillard 1966); however, a negative effect on milk yield of the UG group was observed. Only on three occasions during the trial did goat flock nematode infections increase with some goats being treated; this occurred in September, during lactation peak, and in December and February after lucerne grazing. These results suggest that goat–nematode interaction (Hoste et al. 2010) would have negative effects on goats and their productivity in such a way that, even at moderate burdens, GIN control should not be neglected.

The effect of treatments on milk production shows that, irrespective of the number of parities or type of birth of goats, under the design conditions of the trial, treatment improved milk production and the length of the milking period. Suppressive monthly anthelmintic treatment improved milk production in the first part of the trial, during the period of highest production and after the lucerne grazing period.

The review of Gross, Ryan and Ploeger (1999) showed that in dairy cows with access to pastures, in general there was an economic profit from treatments, with a median increase of 0.63 kg of milk per cow per day over that of untreated cows. In dairy sheep, experimental studies conducted on infected lactating ewes showed a significant effect of nematode infections on production. In two similar studies, sheep infected with 28 000 or 30 000 larvae of *T. circumcincta* showed a 17% or 11.9% reduction, respectively, in milk production compared with worm-free controls (Cruz Rojo et al. 2012; Leyva, Henderson & Sykes 1982). Likewise, a study comparing milk yield in ewes orally infected with 2500 *H. contortus* larvae weekly during pregnancy and lactation reported a marked weight loss and reduction of 23% in milk yield (Thomas & Ali 1983).

On the other hand, references about GIN effect on dairy goats are scarce. Hoste and Chartier (1993) reported that high producer dairy goats responded better to anthelmintic treatment than low producers. Machine-milked goats infected three times with 10 000 *T. colubriformis* and 5000 *H. contortus* third-stage larvae at 50-day intervals showed a general reduction in flock milk yield of 2.5% – 10.0%, whereas in the best producers there was a reduction of 13% – 25%. Studies conducted in Italy involving untreated naturally infected animals and anthelminthic-treated animals showed that milk production was significantly higher in the TGs than in the untreated ones, and treatment led to a persistent increase in milk yield, ranging from 7.4% to 18.5% with respect to control values. In addition, nematode infection might also produce a deteriorating effect on milk quality, since milk from the UG showed 29.9% lower fat content, 23.3% lower protein content and 19.6% lower lactose content than milk from the control group (Rinaldi et al. 2007). In our study, differences between groups were greater than in the study by Rinaldi et al. (2007), with a mean treatment response of TG (399.5 L) representing 117.8 L (41.8 %) higher than UG milk yield (281.6 L).

In our experiment, the length of the milking period was also affected by anthelmintic treatment, as reported by Suarez et al. (2009) with regard to the length of the milking period of untreated dairy sheep. Likewise, in naturally infected goats in France, considering milk production of the whole period, Chartier et al. (2000) observed a significantly longer duration of lactation in the group treated with a high protein diet compared with the group treated with normal protein diet (301.5 vs. 294.9 days) and a similar tendency for total milk yield.

## Conclusion

The present results indicate that *T. colubriformis* and *H. contortus* are the most adverse nematode species for goat production in this region. The results also showed that even under short periods of exposure to a subclinical moderate infection, GINs can have a negative effect on milk production of dairy goats. These results highlight the need for an agreement between veterinarians and researchers on nematode control strategies for preventing milk production losses.
